# Concomitant pancreatic and duodenal metastases 12 years after nephrectomy for renal cell carcinoma: a case report

**DOI:** 10.1093/jscr/rjae276

**Published:** 2024-05-03

**Authors:** Hideharu Tanaka, Shuji Komori, Tomonari Suetsugu, Yoshinori Iwata, Taku Watanabe, Chihiro Tanaka, Narutoshi Nagao, Kei Noguchi, Kenji Hisamatsu, Masaki Katayama, Masahiro Kawai

**Affiliations:** Department of Surgery, Gifu Prefectural General Medical Center, 4-6-1, Noishiki, Gifu, Gifu 500-8717, Japan; Department of Surgery, Gifu Prefectural General Medical Center, 4-6-1, Noishiki, Gifu, Gifu 500-8717, Japan; Department of Surgery, Gifu Prefectural General Medical Center, 4-6-1, Noishiki, Gifu, Gifu 500-8717, Japan; Department of Surgery, Gifu Prefectural General Medical Center, 4-6-1, Noishiki, Gifu, Gifu 500-8717, Japan; Department of Surgery, Gifu Prefectural General Medical Center, 4-6-1, Noishiki, Gifu, Gifu 500-8717, Japan; Department of Surgery, Gifu Prefectural General Medical Center, 4-6-1, Noishiki, Gifu, Gifu 500-8717, Japan; Department of Surgery, Gifu Prefectural General Medical Center, 4-6-1, Noishiki, Gifu, Gifu 500-8717, Japan; Department of Pathology, Gifu Prefectural General Medical Center, 4-6-1, Noishiki, Gifu, Gifu 500-8717, Japan; Department of Pathology, Gifu Prefectural General Medical Center, 4-6-1, Noishiki, Gifu, Gifu 500-8717, Japan; Department of Pathology, Gifu Prefectural General Medical Center, 4-6-1, Noishiki, Gifu, Gifu 500-8717, Japan; Department of Surgery, Gifu Prefectural General Medical Center, 4-6-1, Noishiki, Gifu, Gifu 500-8717, Japan

**Keywords:** renal cell carcinoma, metastatic disease, pancreatic metastasis, duodenal metastasis, surgical resection

## Abstract

In selected patients with metastatic renal cell carcinoma, metastasectomy can achieve prolonged survival. Herein we report a patient with concomitant pancreatic and duodenal metastases occurring 12 years after total right nephrectomy for a renal cell carcinoma. The metastases were successfully treated by a pancreas-sparing duodenectomy and distal pancreatectomy. A 66-year-old man was referred to our hospital with a chief complaint of right upper abdominal pain. He had undergone laparoscopic total right nephrectomy for renal cell carcinoma 12 years before. Enhanced computed tomography showed hypervascular tumors in the pancreatic body and the descending duodenum near the papilla of Vater. Histopathological examination of endoscopic ultrasonography-guided fine needle aspiration cytology specimens revealed metastatic clear cell renal cancer. The patient underwent pancreas-sparing duodenectomy and distal pancreatectomy. He developed a pancreatic fistula after surgery that improved with conservative treatment, and has been free of evidence of recurrence up to 20 months postoperatively.

## Introduction

Renal cell carcinoma (RCC) is the third most frequent malignancy of the genitourinary tract, and it accounts for 3% of all adult malignancies [1]. RCC has a potential to metastasize even 10 years after nephrectomy to almost any site through hematogenous or lymphogenous spread, especially in the form of multiple metastases. Among them, whereas the most common sites are lung, lymph node, bone, liver, and adrenal grands [2], concomitant pancreatic and duodenal metastases are extremely rare. Although molecular targeted drugs have recently been shown to improve the prognosis of patients with metastatic RCC, surgical resection has continued to play an important role in the treatment of metastatic disease [3]. Herein we describe a patient with concomitant pancreatic and duodenal metastases occurring twelve years after total right nephrectomy for RCC.

## Case report

**Figure 1:**
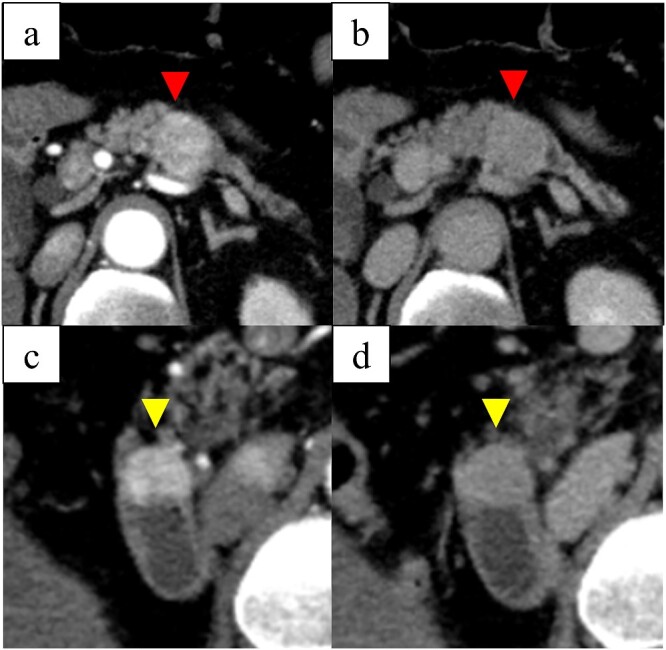
Computed tomography images: (a) arterial-phase axial computed tomography shows a hypervascular tumor in the pancreatic body, (b) the pancreatic tumor was washed out in the venous phase, (c) arterial-phase axial computed tomography shows a hypervascular tumor in the descending duodenum, and (d) the duodenal tumor was washed out in the venous phase.

**Figure 2:**
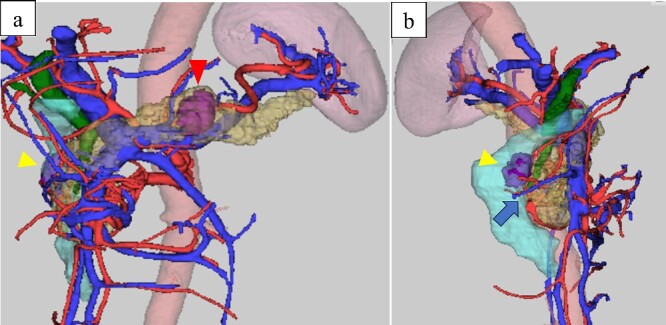
Computed-tomography reconstructed images: (a) frontal view and (b) right lateral view, the papilla of Vater; arrow, duodenal tumor was located near the papilla of Vater.

In 2010, a 54-year-old man underwent laparoscopic total right nephrectomy for RCC at our hospital. Renal cell carcinoma was diagnosed. Based on the criteria of the 8^th^ edition of the UICC the tumor was diagnosed as T1aN0M0 Stage I. The patient did not receive adjuvant chemotherapy after the nephrectomy, and was free of recurrence for 12 years after surgery. In March 2022, he was referred to our hospital with a chief complaint of right upper abdominal pain. Enhanced computed tomography (CT) showed an enlarged gallbladder with findings characteristic of acute cholecystitis. CT also revealed 27- and 20-mm hypervascular tumors in the pancreatic body and descending duodenum near the papilla of Vater. The tumors appeared well enhanced in the arterial phase and washed out in the venous phase (Figs 1 and 2).

The patient’s acute cholecystitis was resolved by percutaneous transhepatic drainage of the gallbladder. The tumors were investigated by upper gastrointestinal endoscopy, which did not find abnormalities in the duodenum, but endoscopic ultrasonography showed a 26-mm well circumscribed tumor in the pancreatic body that had invaded the splenic artery and vein and a 22-mm tumor extending from the duodenum to the head of the pancreas. Endoscopic ultrasonography-guided fine needle aspiration cytology was performed for the pancreatic and duodenal tumors. The histopathological findings revealed metastatic clear cell renal carcinoma. Positron emission tomography was negative for other distant metastases. We decided to perform surgical resection because complete resection of the tumors was possible, and the patient was in good general condition and had been free of disease for a long interval.

**Figure 3:**
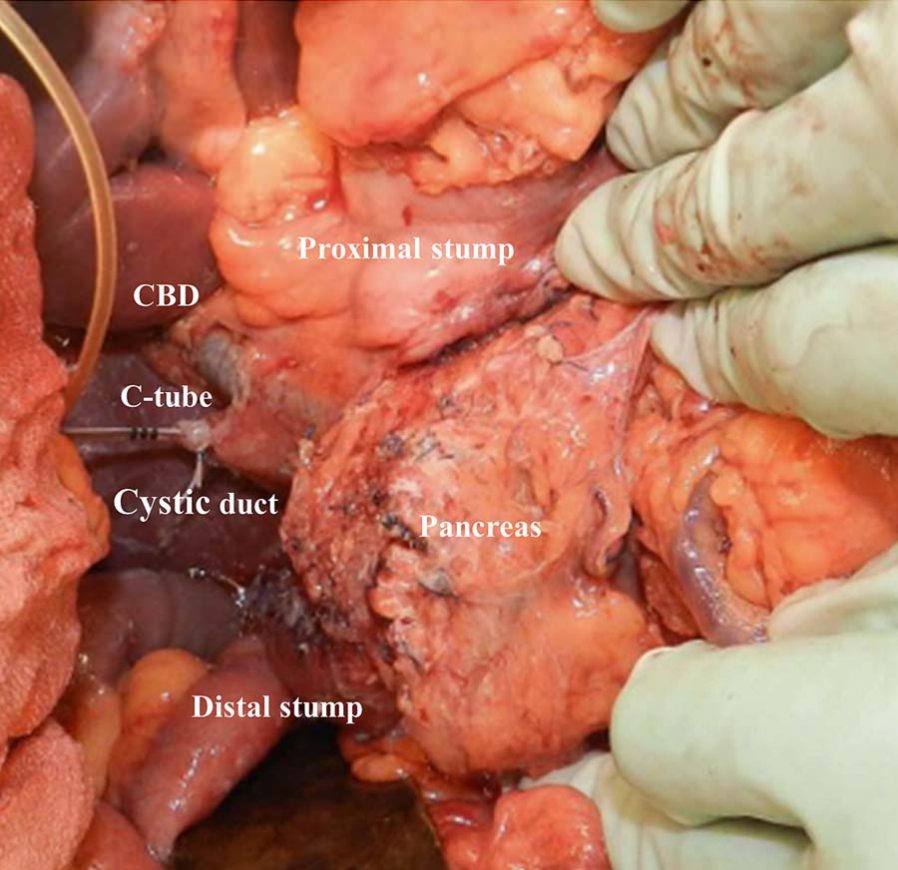
Intraoperative image after pancreas-sparing duodenectomy for duodenal tumor. The bulb and descending duodenum were resected.

**Figure 4:**
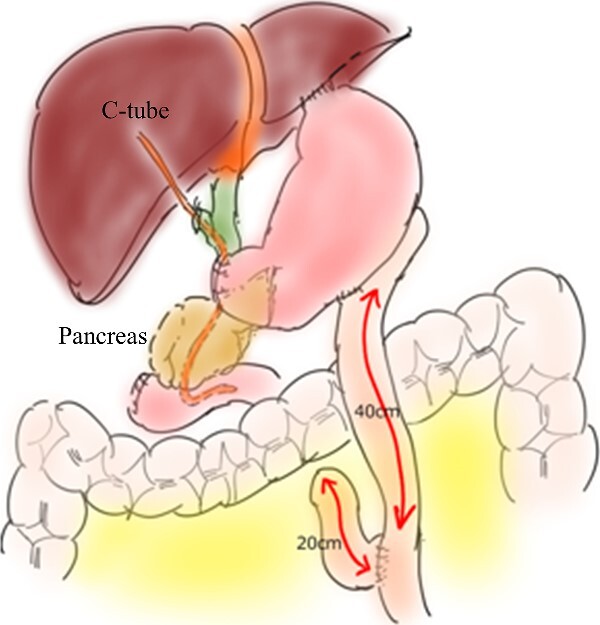
Depiction of reconstruction by the Roux-en Y method.

Intraoperative findings showed that the tumor in the descending duodenum protruded extramurally toward the pancreatic head, and we initially decided to perform a pancreaticoduodenectomy (PD). However, we did not observe invasion of the bile duct or main pancreatic duct and saw that the duodenal tumor could be detached from the pancreas by careful dissection. Thus, we decided to perform a pancreas-sparing duodenectomy. Because the extramural duodenal tumor was located 1 cm from the oral side of the papilla of Vater, resection of the tumor with some of the duodenum could start proximal to the papilla of Vater, and reconstruction of the bile duct and pancreatic duct was not necessary (Fig. 3). We then performed a distal pancreatectomy combined with splenectomy for the tumor in the pancreatic body, and reconstruction by the Roux-en Y method (Fig. 4). The total operative time was 462 min, and the volume of blood loss was 840 ml.

**Figure 5:**
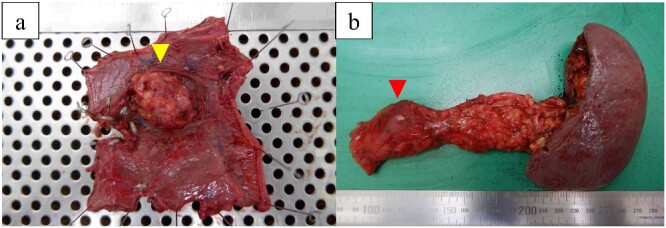
Macroscopic view of the resected specimens. (a) Specimen shows a 30-mm extramural tumor (arrow) in the duodenum. (b) Specimen shows a 30-mm tumor (arrow) in the pancreatic body.

**Figure 6:**
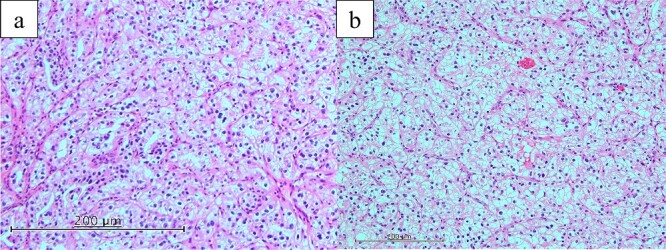
Histopathological findings (hematoxylin and eosin staining). Histopathological examination revealed pancreatic (a) and duodenal (b) metastases of clear cell renal carcinoma.

The excised specimen showed a 30-mm extramural tumor in the duodenum and a 30-mm tumor in the pancreatic body (Fig. 5). Histopathological examinations revealed pancreatic and duodenal metastases of clear cell renal carcinoma (Fig. 6).

The patient developed a pancreatic fistula after surgery that improved with conservative treatment. He was discharged on postoperative Day 42, and has been free of recurrence up to 20 months of follow up.

## Discussion

RCC can metastasize to almost any site in the body, especially in the form of multiple metastases. The most common sites are the lungs (75%), lymph nodes (36%), bones (20%), liver (18%), and adrenal glands [2]. The frequency of pancreatic metastasis from RCC has been reported to be 2.7% [4]. However RCC has been cited as the most common primary malignancy that produces solitary pancreatic metastases; 61.7% to 70.5% of solitary pancreatic metastases are RCCs [5]. On the other hand, the frequency of duodenal metastases from RCCs has been reported to be 0.16% [6].

Whereas most pancreatic metastases are asymptomatic, most patients with duodenal metastases from RCC present with gastrointestinal (GI) bleeding or obstructive symptoms. However, our case was atypical because GI bleeding was absent.

RCC also can metastasize >10 years after nephrectomy. The mean duration from nephrectomy to pancreatic metastasis has been reported to range from 9.8 to 11.2 years [7, 8]. Noguchi *et al.* [4] reported that the rates of recurrence at typical sites decreased and recurrence in retroperitoneal organs such as the pancreas increased in a time-dependent manner.

Treatments for metastatic RCC include molecularly targeted drugs, which have led to an improved prognosis compared to cytokine therapy. However, obtaining a complete response with molecularly targeted drugs alone is rare. In selected patients with metastatic RCC who are in good general condition and free of disease over a long period of time, the disease can be controlled by local therapies such as metastasectomy, which can further prolong their survival [9].

Systematic reviews have consistently suggested that complete metastasectomy for metastatic RCC, including resections of multiple metastases, was beneficial for overall survival and cancer-specific survival. They have also suggested that with the exception of metastases to brain and bone, complete metastasectomy is the most appropriate local therapy [3, 10].

Depending on the site of the tumor in relation to the ampulla of Vater, the surgical methods reported for duodenal metastases from RCC range from duodenectomy to pancreaticoduodenectomy [11, 12]. For metastases to the pancreas in relation to the site of the tumor, standardized pancreatic resections have included pancreaticoduodenectomy, distal pancreatectomy, and total pancreatectomy [12].

To our best knowledge, with the inclusion of our case, there have been six published cases reports on concomitant duodenal and pancreatic metastatic RCCs [11–14] (Table 1). Surgery was performed in four cases, and in all four cases, no signs of recurrence have been reported. For our patient, if a pancreaticoduodenectomy that also removed the tumor in the pancreatic body had been performed, the residual pancreas would have been extremely small. Since we were concerned that the result would have greatly affected the postoperative nutritional status, we performed a pancreas-sparing duodenectomy and distal pancreatectomy. To date, the patient has neither required insulin injections nor medication for diabetes after surgery, and has maintained good nutritional status without developing recurrent disease.

**Table 1:** Reported cases of concomitant pancreatic and duodenal metastases from renal cell carcinoma

No.	Authors	Year	Sex	Age (yr)	Primary location	Symptoms	Time from initial surgery to metastasis (yr)	Treatment	Outcome
1	Hashimoto	1996	M	68	Left kidney	GI bleeding	11	Total pancreatectomy with duodenectomy	Alive (18 months)
2	Segawa	2006	M	52	Right kidney	Anemia, vertigo	13	Interferon	Dead
3	Espinoza	2014	M	58	Right kidney	GI bleeding	12	Pancreaticoduodenectomy	Alive
4	Espinoza	2014	F	66	Right kidney	Abdominal pain	4	Pancreaticoduodenectomy	Alive
5	Lu	2021	F	76	Right kidney	Jaundice	17	Symptomatic treatment	Alive (20 months)
6	Our patient	2023	M	66	Right kidney	Abdominal pain	12	Pancreas-sparing duodenectomy, Distal pancreatectomy	Alive (20 months)

In conclusion, especially for patients with clear cell RCC, follow-ups longer than 10 years are needed. Rigorous considerations that include the metastatic sites and general condition of the patients are also needed when choosing the optimal operative procedures for pancreatic and duodenal metastases.

## Data Availability

Data can be obtained by emailing the corresponding author.
